# The subunit of RNA N6-methyladenosine methyltransferase OsFIP regulates early degeneration of microspores in rice

**DOI:** 10.1371/journal.pgen.1008120

**Published:** 2019-05-22

**Authors:** Fan Zhang, Yu-Chan Zhang, Jian-You Liao, Yang Yu, Yan-Fei Zhou, Yan-Zhao Feng, Yu-Wei Yang, Meng-Qi Lei, Mei Bai, Hong Wu, Yue-Qin Chen

**Affiliations:** 1 Guangdong Provincial Key Laboratory of Plant Resources, State Key Laboratory for Biocontrol, School of Life Sciences, Sun Yat-Sen University, Guangzhou, P. R. China; 2 Medical Research Center, Sun Yat-Sen Memorial Hospital, Sun Yat-Sen University, Guangzhou, China; 3 State Key Laboratory for Conservation and Utilization of Subtropical Agro-bioresources, College of Life Sciences, South China Agricultural University, Guangzhou, P. R. China; The University of Adelaide, AUSTRALIA

## Abstract

N^6^-Methyladenosine (m^6^A) RNA methylation plays important roles during development in different species. However, knowledge of m^6^A RNA methylation in monocots remains limited. In this study, we reported that *OsFIP* and *OsMTA2* are the components of m^6^A RNA methyltransferase complex in rice and uncovered a previously unknown function of m^6^A RNA methylation in regulation of plant sporogenesis. Importantly, *OsFIP* is essential for rice male gametogenesis. Knocking out of *OsFIP* results in early degeneration of microspores at the vacuolated pollen stage and simultaneously causes abnormal meiosis in prophase I. We further analyzed the profile of rice m^6^A modification during sporogenesis in both WT and OsFIP loss-of-function plants, and identified a rice panicle specific m^6^A modification motif “UGWAMH”. Interestingly, we found that *OsFIP* directly mediates the m^6^A methylation of a set of threonine protease and NTPase mRNAs and is essential for their expression and/or splicing, which in turn regulates the progress of sporogenesis. Our findings revealed for the first time that *OsFIP* plays an indispensable role in plant early sporogenesis. This study also provides evidence for the different functions of the m^6^A RNA methyltransferase complex between rice and Arabidopsis.

## Author summary

N^6^-Methyladenosine (m^6^A) is the most abundant internal modification of eukaryotic mRNA, and m^6^A mRNA methylation affects almost every stage of mRNA metabolism. However, the components of the m^6^A methyltransferase complex and their functions in monocots are completely unknown. In this study, we identified the components of the m^6^A RNA methyltransferase complex in rice, and uncovered a hitherto unknown function of m^6^A RNA methylation in regulating early microspore apoptosis. We also systematically analyzed the characteristics of m^6^A modification during sporogenesis for the first time, and revealed the sporogenesis stage-specific distribution of m^6^A peaks along genes and the specific modification motif in rice, which are different from that of other species and other developmental stages. The target genes of m^6^A methyltransferase complex member *OsFIP* were also identified in this study. Given the important roles of posttranscriptional mRNA regulation in gene expression and sporogenesis in plants, the findings of this study should stimulate more studies exploring the role of plant m^6^A methyltransferase and other components.

## Introduction

N^6^-methyladenosine (m^6^A) represents the most abundant internal modification of eukaryotic mRNA and accounts for more than 80% of all RNA base methylations in various species. m^6^A mRNA methylation affects almost every stage of mRNA metabolism. The deposition of m^6^A is achieved through a multicomponent methyltransferase complex [1]. In mammals, methyltransferase-like 3 (METTL3) is responsible for methylation activity [2]. METTL14 and Wilms’ tumor 1-associating protein (WTAP) are other components of the m^6^A methyltransferase complex that have also been identified [3, 4]. WTAP associates with the METTL3-METTL14 core complex and facilitates METTL3-MELLT14 complex translocation to nuclear speckles, and this activity is required for the efficient methylation of mRNA [5, 6].

Most of the progress in elucidating plant m^6^A methylation machineries and their functions have been achieved in Arabidopsis [7–9]. In Arabidopsis, the ortholog of METTL3 is mRNA adenosine methylase (MTA). The inactivation of MTA results in reduced m^6^A mRNA methylation and a failure of the developing embryo to progress past the globular stage [5, 10]. AtFIP37 is the ortholog of mammalian WTAP in Arabidopsis. *AtFIP37* knockout mutants show an embryo-lethal phenotype that is caused by a strong delay in endosperm development and embryo arrest [11]. Moreover, a recent study showed that AtFIP37 plays an indispensable role in determining shoot stem cell fate in Arabidopsis [12]. All together, these studies indicate that the m^6^A methyltransferase components have unique functions during embryo development, shoot stem cell fate and root growth in Arabidopsis.

However, the components and functions of m^6^A methyltransferases in monocot species have not been reported. Here, we identified the components of the m^6^A methyltransferase complex in rice and uncovered a previously unknown function of m^6^A methylation in the regulation of pollen development. We revealed that OsFIP and OsMTA2 are the orthologues of Arabidopsis FIP37 and MTA, respectively. They interact with each other and both of them are required for mRNA methylation. The unique function of OsFIP was further revealed in this study. OsFIP is essential for early sporogenesis. Loss of function of *OsFIP* disrupts the m^6^A modifications of threonine protease and NTPase genes during sporogenesis by directly binding to them and leads to microspores being degenerate at the early microspore stage. OsFIP also affects both the chromosomes and the cytoplasmic components of microspore mother cells (MMCs) during prophase I. These findings revealed the essential roles of OsFIP in rice sporogenesis and fertility.

## Results

### OsFIP and OsMTA2 are the subunits of RNA N6-methyladenosine methyltransferase in rice

To identify the rice components of the m^6^A methyltransferase complex and explore their functions, we first searched for homologs of the m^6^A methyltransferase complex in mammals and Arabidopsis. Five rice proteins were predicted to be the m^6^A methyltransferase components: OsMTA2 (LOC_Os02g45110), which is 57.2% identical to AtMTA (**S1A Fig**); OsFIP (LOC_Os06g27970), which is 59.09% identical to AtFIP37 (**S1B Fig**); and OsMTA1 (LOC_Os01g16180), OsMTA3 (LOC_Os03g05420) and OsMTA4 (LOC_Os10g31030), which are 54.86%, 43.15% and 48.53% identical to AtMTB, respectively (**S1C Fig**). Although the functions of these five proteins are unknown, their functional regions are highly conserved (**S1A** and **S1C Fig**).

**Fig 1:**
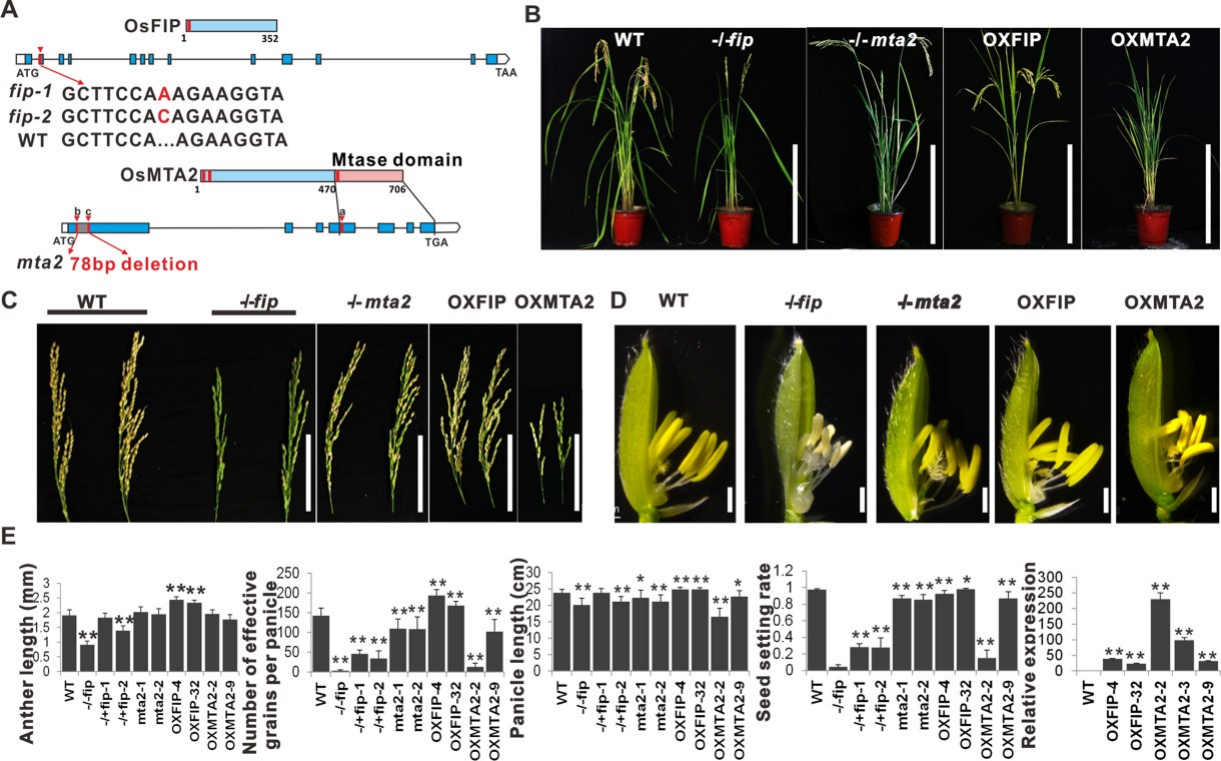
*OsFIP* and *OsMTA2* are required for reproductive development. (**A**) Schematic of the *OsMTA2* and *OsFIP* knockout mutants generated by CRISPR-Cas9. The red arrowheads indicate the gRNA target sites. (**B**) Gross morphology of wild-type, *fip*, *mta2*, OXFIP and OXMTA2 transgenic plants. Scale bar, 40 cm. (**C**) Panicles of WT, *fip*, *mta2*, OXFIP and OXMTA2 plants. Scale bars, 10 cm. (**D**) Spikelets of WT, *fip*, *mta2* OXFIP and OXMTA2 plants. Scale bars, 1 mm. (**E**) Panicle lengths, seed numbers, seed-setting rates, anther lengths and relative expression of genes of different transgenic lines. Values shown are the means ± s.d. (n > 15 plants). Significant differences were identified using Student’s *t*-test.

To verify whether these five proteins are the subunits of m^6^A RNA methyltransferase in rice, we constructed their knockout mutant lines using CRISPR-Cas9, respectively (named *mta2*, *fip*, *mta1*, *mta3* and *mta4*). For the *mta2* mutant, the gRNA target was first designed to target the start position of the predicted Mtase domain (the fourth exon, **Fig 1A**). However, no homozygous *OsMTA2* knockout line with a reading frame shift mutation was obtained despite generating *OsMTA2* knockout mutants twice and screening more than 300 plants. We speculated that *OsMTA2* is indispensable for rice callus differentiation. We then designed two gRNAs targeting the first exon of *OsMTA2*, and no mutant line with a reading frame shift mutation was obtained; only two homozygous lines with a 28 amino acids deletion in the noncatalytic region or 28 amino acids substitution respectively were obtained and were used for further study (**Fig 1A** and **S2A Fig**). For the *fip* mutant, the gRNA was designed to target the second exon. We identified several homozygous *fip* mutant lines that had reading frame shifts (**Fig 1A** and **S2B Fig**). For the *mta1*, *mta3* and *mta4* mutants, the gRNA targets were designed to target the first exon (**S2C Fig**).

We next performed dot blot analyses to compare the total m^6^A levels in RNA from all the knockout mutant lines. As expected, knocking out of *OsFIP* or *OsMTA2* dramatically reduced m^6^A levels (**S3A** and **S3B Fig**), indicating that *OsFIP* and *OsMTA2* are required for global m^6^A RNA methylation in rice. However, no effects on the total m^6^A levels were observed in the *OsMTA1*, *OsMTA3* and *OsMTA4* knockout lines (**S3C Fig**). We also constructed transgenic plants overexpressing *OsFIP* (OXFIP) or *OsMTA2* (OXMTA2) to investigate the regulatory roles of high levels of these proteins on m^6^A abundance and plant growth (**Fig 1E**). Consistently, m^6^A levels were slightly increased in the OXMTA2 plants and in the OXFIP lines (**S3A Fig**). We next examined whether the interaction between OsMTAs and OsFIP occurs *in vivo* using yeast two-hybrid experiments and bimolecular fluorescence complementation (BiFC). The results clearly showed that OsMTA2 interacts with OsFIP in both yeast and rice nuclei (**S3D** and **S3E Fig**). However, OsMTA1, OsMTA3 and OsMTA4 did not interact with OsMTA2 or OsFIP (**S3E Fig**). Together with the effects of these five proteins on the m^6^A levels in rice, we proposed that OsMTA2 and OsFIP are the subunits of RNA N6-methyladenosine methyltransferase in rice but that OsMTA1, OsMTA3 and OsMTA4 might not be components of the complex.

### Phenotypic analysis of *OsFIP* and *OsMTA2* mutants revealed a unique function of *OsFIP* in sporogenesis

Next, to investigate the functional relevance of *OsFIP* and *OsMTA2* in rice development, we performed phenotypic analyses of the knockout mutant lines (*mta2* and *fip*) and the overexpression lines (OXMTA2 and OXFIP) (**Fig 1B**). In the vegetative stage, the phenotypes of the four mutant plants appear normal and similar to that of the wild-type plants (**S3F Fig**), only the tiller number of homozygous *fip* plants (~1.4 tillers per plant) was less than that of WT plants (~4.7 tillers per plant) (**S3G Fig**). However, in the late stage of reproductive development, the *fip* plants were almost totally sterile and presented shortened panicles and anthers, and decreased effective seed number compared with that of the wild-type (WT) plants, whereas OXFIP have longer anthers, longer panicles, higher seed numbers and seed setting rates than WT plants (**Fig 1C–1E**). For the *mta2* and OXMTA2 plants, the panicles length, fertility and effective seed number were also reduced compared with those of the WT plants but were higher than those of the *fip* plants (**Fig 1C–1E**).

**Fig 2:**
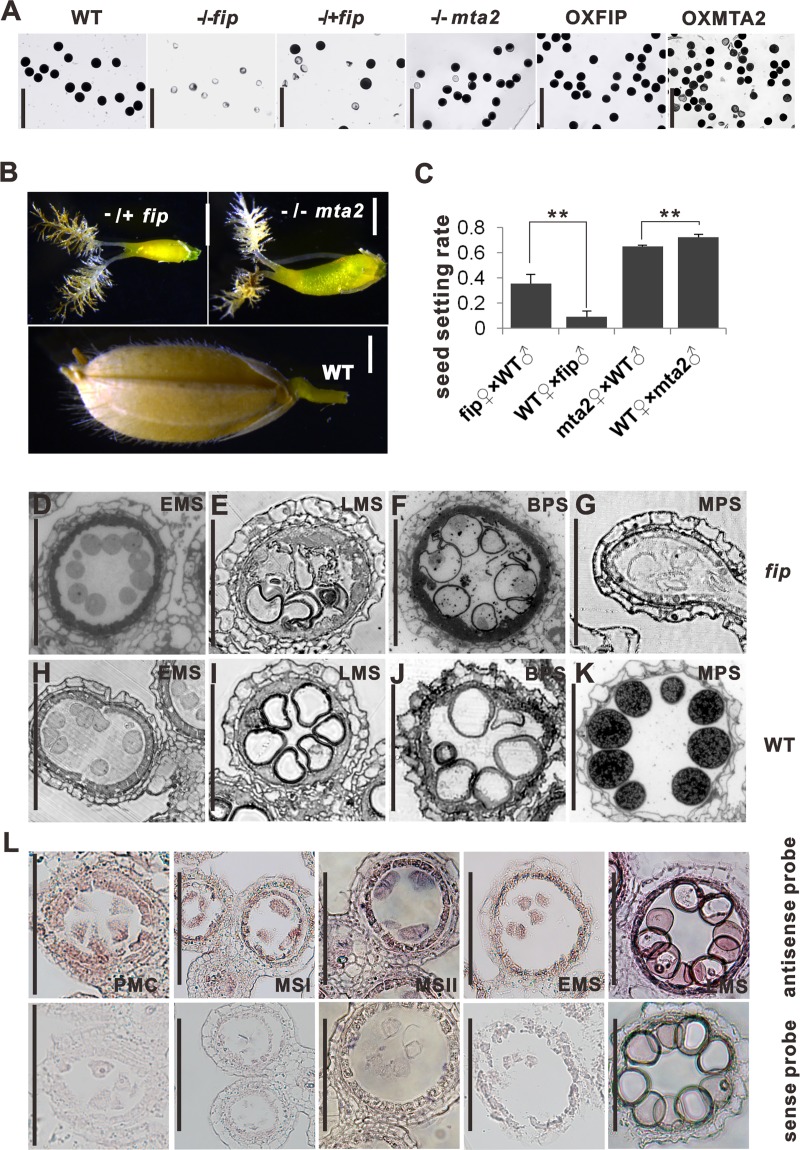
*OsFIP* and *OsMTA2* regulate different stages of male reproductive development. (**A**) Mature pollen grains at stage 12 stained for starch with I_2_-KI. Scale bars, 100 μm. (**B**) Seeds of WT, *fip* and *mta2* at 21 days after flowering. Scale bars, 0.7 mm. (**C**) The seed setting rates of the mutants crossed with WT plants. Values shown are the means ± s.d. (n = 4 plants). Significant differences were identified using Student’s *t*-test. (**D-K**) Transverse semithin sections of homozygous *fip* anthers at stages 9 (**D**), 10 (**E**) 11–12 (**F**) and 12 (**G**) from left to right panels; WT anthers at stages 9 (**H**), 10 (**I**), 11–12 (**J**) and 12 (**K**) from left to right panels. Scale bars, 100 μm. (**L**) In situ hybridization of *OsFIP* mRNAs from stage 6, 7, 8, 9 and 10 anthers from left to right panels. The sense probes of *OsFIP* mRNAs were used as negative controls. Scale bars, 100 μm. PMC, pollen mother cell; MSI, meiosis I; MSII, meiosis II; EMS, early microspore stage; LMS, late microspore stage; BPS, binucleate pollen stage; MPS, mature pollen stage.

To understand what caused the sterile phenotypes of *mta2* and *fip*, we examined the pistil and stamen structures of the four mutants mentioned above. As shown in S**3**D Fig, all of the mature pistils (n>60 pistils for each line) of the mutants showed normal embryo sac development (**S3H Fig**), suggesting that the low setting rates in *mta2* and *fip* might not be associated with pistil development. We then examined the pollen grains of the transgenic plants, and the results showed that the *fip* anthers had very few pollen grains, and 84.8% of the existing pollen grains lacked starch, but *mta2* and OXMTA2 only have a few abortive pollen grains (17.1% for *mta2*, 27.5% for OXMTA2) (**Fig 2A)**, indicating that OsFIP plays an essential role in microspore development.

It is generally considered that seed development also affects plant seed-setting rates. We therefore analyzed the seeds of the mutants at 21 days after pollination to investigate the seed development process. In the abortive seeds of homozygous *mta2* panicles and in OXMTA2 panicles, approximately 80% and 91.0%, respectively, of the ovaries were pollinated but did not fully develop (**Fig 2B** and **S3H Fig)**. However, very different from those in the *mta2* mutants, almost all of the ovaries in the homozygous *fip* plants at 21 days after pollination appeared unpollinated (**Fig 2B** and **S3H Fig**). To further confirm whether embryo development is normal or not in *mat2* and *fip* mutants, we crossed *fip* and *mta2* plants with WT plants. As expected, when we used *mta2* as the female parent the seed setting rate was lower than when using *mta2* as the male parent (**Fig 2C**). However, when we used *fip* as the female or male parent, the seed setting rates were also low, and much lower when using *fip* as male parent (**Fig 2C**). Together, these results indicated that OsMTA2 has a conserved role in regulating embryo development between rice and Arabidopsis, and OsFIP is required for both sporogenesis and embryo development, and the failed sporogenesis might be the dominant reason for the decreased seed setting rate in *fip* plants. There is no prior report about the role of RNA m6A methyltransferase in regulating sporogenesis in plants [5, 10–12], and thus, in the following experiments, we focused mainly on OsFIP.

### *OsFIP* regulates the early degeneration of microspores at the vacuolated pollen stage

To examine how OsFIP regulates microspore development, we obtained semithin sections for observing anther development from stage 6 [13] (MMC differentiation stage) to stage 12 (late binucleate pollen stage) comparing WT and *fip* mutants (**Fig 2D–2K, S3I** and **S3J Fig)**. The semi sections showed that no obvious abnormity in *fip* anthers at pollen mother cell stage, meiosis stage, and tetrad stage (**S3I Fig**). However, at vacuolated stage the microspores had irregular shapes and some debris in anther lobes (**Fig 2E**), at binucleate pollen stage the pollen degeneration became severe and had unknown particles in anther lobes (**Fig 2F**), and at mature stage the pollens lost their cytoplasm and were completely collapsed (**Fig 2G**). In the heterozygous *fip* plants, approximately half of the microspores could not mature after heading (**S3J Fig**). The results showed that the degeneration of microspores might begin from the vacuolated stage. We further examined the spatial expression pattern of *OsFIP*. As shown in **Fig 2L**, *OsFIP* expression was detected in MMCs, tapetal cells and microspores from stage 6 to stage 9, and the signals intensified in vacuolated microspores and tapetal cells at stage 10, indicating that OsFIP could involve in microspore developmental process.

**Fig 3:**
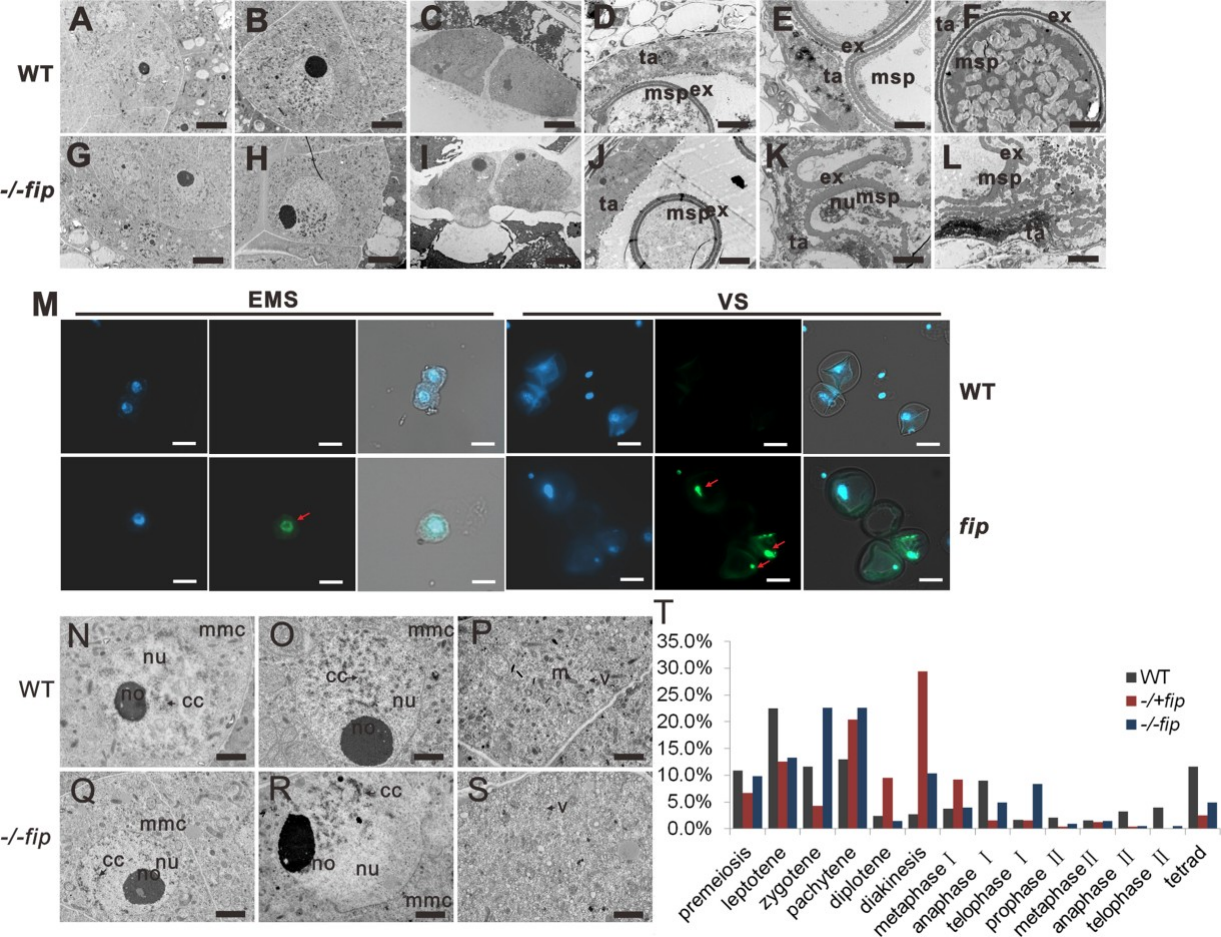
Histological analysis of WT and homozygous *fip* MMCs and microspores. (**A**) and (**G**) show the MMCs of WT (**A**) and *fip* (**G**) at the leptotene stage. (**B**) and (**H**) show the MMCs of WT (**B**) and *fip* (**H**) at the pachytene stage. (**C**) and (**I**) showed the MMCs of WT (**H**) and *fip* (**K**) at meiosis stage. (**D**) and (**J**) showed the microspore and tapetum of WT (**D**) and *fip* (**J**) at early microspore stage. (**E**) and (**K**) showed the microspore and tapetum of WT (**E**) and *fip* (**K**) at late microspore stage. (**F**) and (**L**) showed the microspore and tapetum of WT (**F**) and *fip* (**L**) at mature stage. Scale bars, 5μm. msp, microspores; ex, exine; ta, tapetum. (**M**) TULNEL assay of the WT and *fip* pollen grains at early microspore stage (EMS) and vacuolated stage (VS). Scale bars, 20 μm. (**N**) and (**Q**) show the nucleus of WT (**A**) and *fip* (**D**) MMCs at the leptotene stage. (**O**) and (**P**) show the nucleus (**O**) and cytoplasm (**P**) of WT MMCs at the pachytene stage. (**R**) and (**S**) show the nucleus (**R**) and cytoplasm (**S**) of *fip* MMCs at the pachytene stage. Scale bars, 2 μm. (**T**) Frequency of WT and *fip* MMCs at various meiotic stages in all the anthers analyzed.

To investigate which process of microspore development OsFIP might affect, we compared the ultrastructure between WT and homozygous *fip* anthers from stage 6 to stage 12 (**Fig 3A–3L**). The most obvious abnormal phenotype of *fip* plants appeared at the vacuolated pollen stage. Almost all of the homozygous *fip* microspores degenerated at the vacuolated pollen stage (stage 10, late microspore stage) (**Fig 3K**). At this stage, the WT microspores have a clear single nucleus (**Fig 3E**); however, the homozygous *fip* microspores have no nucleus (**Fig 3K**), and 37.8% of the heterozygous *fip* microspores have no nucleus (**S4Q Fig**). **S4Q** and **S4R Fig** shows the number of nuclei of WT and *fip* microspores during the late micropore stage (LMS), late binucleate pollen stage (LBPS) and mature pollen stage (MPS). However, at the LMS, the nuclei and the pollen exines (ex) of the *fip* microspores (msp) were undergoing degeneration (**Fig 3K**) and degraded completely at the mature stage (**Fig 3L**). We also analyzed the heterozygous *fip* microspores. At stage 11, most of the WT microspores underwent the first mitotic division and generated a smaller generative cell and a larger vegetative cell; however, in the heterozygous *fip* plants, 23.2% of the *fip* microspores had only one nucleus, and 27.5% of the *fip* microspores had no nucleus. At stage 12, the WT microspores underwent a second mitosis and divided into two sperm cells, and the mature pollen grain contained three nuclei; however, 5.4% of the heterozygous *fip* microspores possessed one nucleus, 31.9% possessed no nucleus, and 54.0% of heterozygous *fip* microspores could not accumulate starch (**S4Q** and **S4R Fig**). These results showed that *OsFIP* affects the early apoptosis of the microspores at the vacuolated pollen stage.

To further validate whether the pollen grains degenerated from the vacuolated stage, we have performed TULNEL assay to the *fip* pollen grains at early microspore stage and vacuolated stage. A few signal was first detected in *fip* pollens at early microspore stage (23.3%), and was obvious in the nucleus of *fip* pollens at the vacuolated pollen stage (48.6%) which was not detected in WT samples (2.7% and 5.3%, respectively) (**Fig 3M**), indicating that *fip* pollen grains began to degenerate from early microspore stage, and the degeneration became obvious at vacuolated stage.

To identify whether the degeneration of microspores were caused by failed meiosis, we then investigated the meiosis processes of *fip* plants. Abnormalities were observed at early meiosis prophase. For example, at the leptotene stage (**Fig 3N**), WT chromosome condensation starts, and scattered chromosomes were observed, indicating DNA replication. At the pachytene stage (**Fig 3O**), homologous chromosome synapsis is complete, and the paired chromosomes thicken and regularly adhere to the nucleolus. However, in the *fip* MMCs, the chromosomes are less condensed at the leptotene stage (**Fig 3Q**), and the distribution of chromosomes are abnormal at the pachytene stage; the chromosomes could not adhere to the nucleolus but were always arranged in one corner (**Fig 3R**). In addition, we also observed clear differences between the cytoplasm of the WT and the *fip* MMCs. At the pachytene stage, vacuoles or autophagosome-like organelles frequently appeared in the *fip* MMCs but rarely appeared in the WT MMCs; other organelles were indistinct in the *fip* MMCs, whereas other organelles, especially an abundance of mitochondria, were visible in the cytoplasm of the WT MMCs (**Fig 3P and 3S**). These results showed that the loss of function of *OsFIP* disrupts both the chromosomes and the cytoplasmic components of MMCs. We also observed a slight arrest of both homozygous and heterozygous *fip* MMCs at prophase I (**Fig 3T** and **S4A Fig**) and a small portion of *fip* MMCs that were abnormal between WT and *fip* MMCs (**S4B–S4P Fig**). For example, in *fip* plants, more or less than 12 bivalents occasionally appeared, the bivalents of a small portion of MMCs could not align at the equatorial plate (**S4L–S4P Fig**), and sometimes chromosome bridges or chromosome fragmentation appeared in the *fip* MMCs (**S4J** and **S4K Fig**). It has been considered that early microspores apoptosis are frequently caused by failed meiosis [14–17]. In *fip* plants, the tetrad at stage 8 and the early microspores at stage 9 seem normally developed (**Figs 2D, 3I–3J** and **S3I Fig**), and *fip* microspores could go through meiosis stage, although the meiosis events were affected in *fip* MMCs (**Fig 3N–3S** and **S4A–S4P Fig**). However, we could observe degraded pollen grains from the vacuolated stage at stage 10 (**Figs 2E and 3K**). Thus we speculated that OsFIP is essential for microspore development from vacuolated stage to mature stage. Together, the data indicated that OsFIP is important for sporogenesis in rice, and loss of function of OsFIP mainly induces microspores degeneration at the vacuolated pollen stage and partially disturbs the meiosis events.

### *OsFIP* is indispensable for m^6^A mRNA modification during early sporogenesis

**Fig 4:**
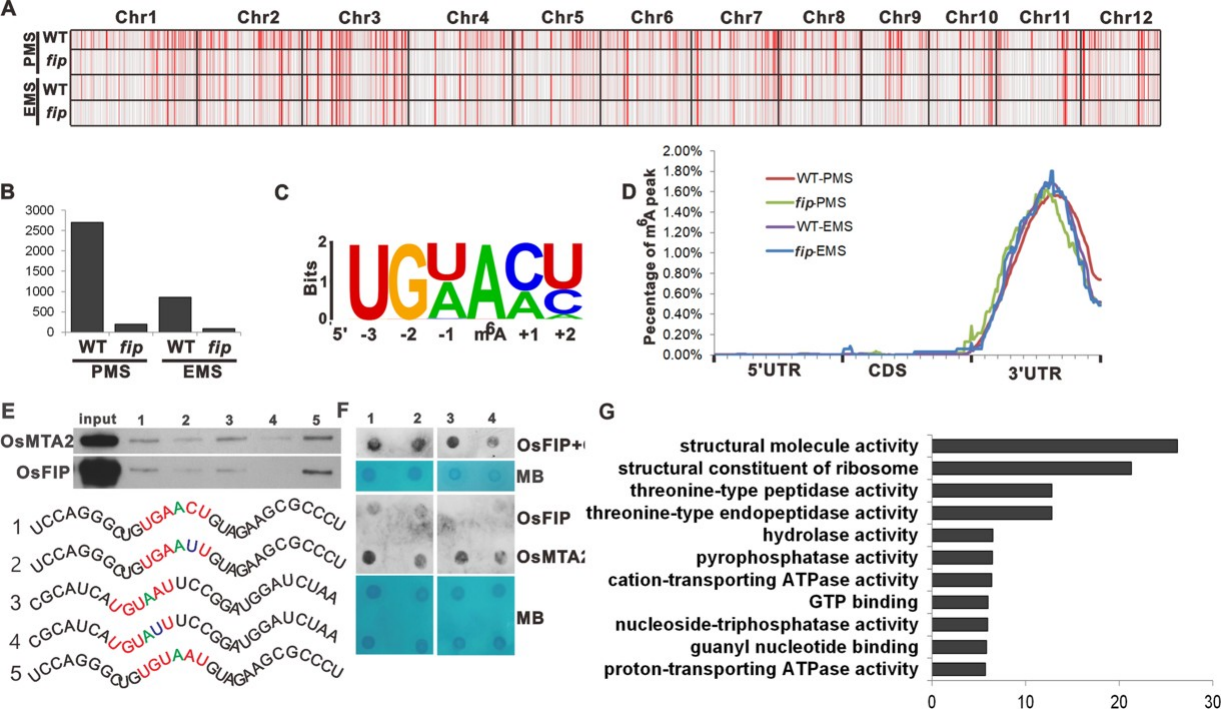
Overview of Distribution of m^6^A modification peaks along mRNA and chromosome, and the methyltransferase activity of OsMTA2 and OsFIP through motifs. (**A**) Distribution of m^6^A peaks along chromosomes of callus and leaf tissues. PMS, pollen mother cell stage; EMS, early microspore stage. (**B**) The number of m6A peaks of WT and *fip* panicles during PMS and EMS. (**C**) The UGWAMH conserved sequence motif for m6A-containing peak regions. (**D**) Distribution of m^6^A peaks in transcript segments divided into 5’UTR, CDS, and 3’UTR in wild-type and *fip* panicles at PMS and EMS. (**E**) The in vitro binding of GFP-tagged OsFIP or OsMTA2 to different RNA probes (numbered probe 1–5) with the rice panicle specific motif UGUAAU or the mammal motif GAACU or the mutated motif. (**F**) The in vitro RNA N6-adenosine methylation activities of GFP-tagged OsFIP or OsMTA2 as well as the combination of OsFIP and OsMTA2 were tested using different RNA probes. MB, methylene blue staining (as loading control). (**G**) The top enriched GO terms of the differentially m^6^A modified genes in *fip* panicles.

Finally, we investigated the underlying mechanism of the *OsFIP* regulation of sporogenesis. To understand how *OsFIP* contributes to the global m^6^A modification during pollen grains development, we performed m^6^A-sequencing on both WT and *fip* anthers at PMS and EMS and compared their transcriptome-wide m^6^A methylomes during meiosis (SRA: accession no. SRR8934214, SRR8934213, SRR8934212 and SRR8934211). A total of 381.9 million reads were generated from twelve libraries and uniquely aligned to the rice genome (STAR). We used exomePeak to detect the m^6^A peaks with an estimated p-value <0.01. Our data revealed 2699 and 863 putative high-confidence m^6^A peaks within 1909 and 568 genes from the WT PMS anthers and the WT EMS anthers, respectively (**Fig 4A and 4B)**, indicating that the m^6^A modification was decreased after pollen mother cell meiosis. However, only 197 and 91 putative high-confidence m^6^A peaks within 112 and 49 genes were detected from the *fip* PMS anthers and the *fip* EMS anthers, respectively (**Fig 4A and 4B)**, indicating that most of the m^6^A modifications during meiosis were *OsFIP* dependent.

The conserved m^6^A modification motif during sporogenesis was then analyzed in both WT and *fip* panicles. The m^6^A modification motif “UGWAMH” (W = U or A; M = C or A; H = U, A or C) was significantly overrepresented (P<10^−146^) in WT panicles (**Fig 4C)**, which is different from the conserved motif in Arabidopsis “RRACH” (R = G or A; H = A, C or U) [18] and is also different from that of rice callus “RAGRAG” but is similar to that of rice leaf “UGUAMM” [19], indicating that the recognition of m^6^A modification sites during sporogenesis might be different from other developmental stages. In the *fip* panicles no conserved motif was identified from the remaining m^6^A modifications.

Next, we analyzed the distribution of m^6^A peaks in genes in rice. The m^6^A peaks were greatly enriched at the middle of the 3’UTRs in both WT panicles and *fip* panicles at PMS and EMS (**Fig 4D)**. Interestingly, the m^6^A modification distribution pattern during rice sporogenesis was different from that of rice leaf and callus, in which most of the m^6^A peaks are localized at the beginning of 3’UTRs and CDS. The difference might be related to the variant regulatory roles of m^6^A modification during different developmental stages. To identify the distribution of m^6^A peaks on mRNA abundance, we compared the expression levels of the mRNAs with fewer m^6^A modifications in the *fip* panicles between WT and *fip* panicles. Approximately 58% of the differentially expressed mRNAs, which also have fewer m^6^A modifications in the *fip* panicles, were upregulated in the *fip* panicles, and approximately 6% of them were alternatively spliced in the *fip* panicles (**S4S–S4T Fig**).

To verify the methyltransferase activity of OsMTA2 and OsFIP through this motif, we then tested N6-adenosine methylation activity. Binding efficiency for GFP-tagged OsFIP or OsMTA2 as well as the combination of OsFIP and OsMTA2 were tested using different RNA probes (numbered probe 1 to 5) with the rice panicle specific motif UGUAAU or the mammal motif GAACU or the mutated motif (**Fig 4E and 4F**). The methylation and binding of RNA probes was measured by immunoblotting with the m^6^A antibody and pulldown assays. Five probes were synthesized and applied: probe 1 has the known m6A motif in mammals “GAACU”, probe 3 and probe 5 have rice panicle specific motif “UGUAAU”, respectively, probe 2 has mutated motif “UGAAUU” and probe 4 has mutated motif “UGUAUU” (**Fig 4E**). As expected, the binding efficiency of OsFIP and OsMTA2 to the rice pollen specific motif or mammal motif was higher than those of mutated motifs (**Fig 4E**). Moreover, OsMTA2-OsFIP complexes or OsMTA2 itself in vitro exhibited m^6^A methyltransferase activity against rice pollen specific motif and mammal motif, but displayed less methyltransferase activity towards the mutant motif (**Fig 4F**). Thus, our data demonstrated that the motif identified in rice panicles was indeed a substrate of rice methyltransferase. Thus, OsFIP is essential for most of the m^6^A modification during sporogenesis by recognizing a rice panicle specific motif “UGWAMH”.

### OsFIP directly binds to the mRNA of threonine protease and NTPase and mediates their m^6^A modification and expression

**Fig 5:**
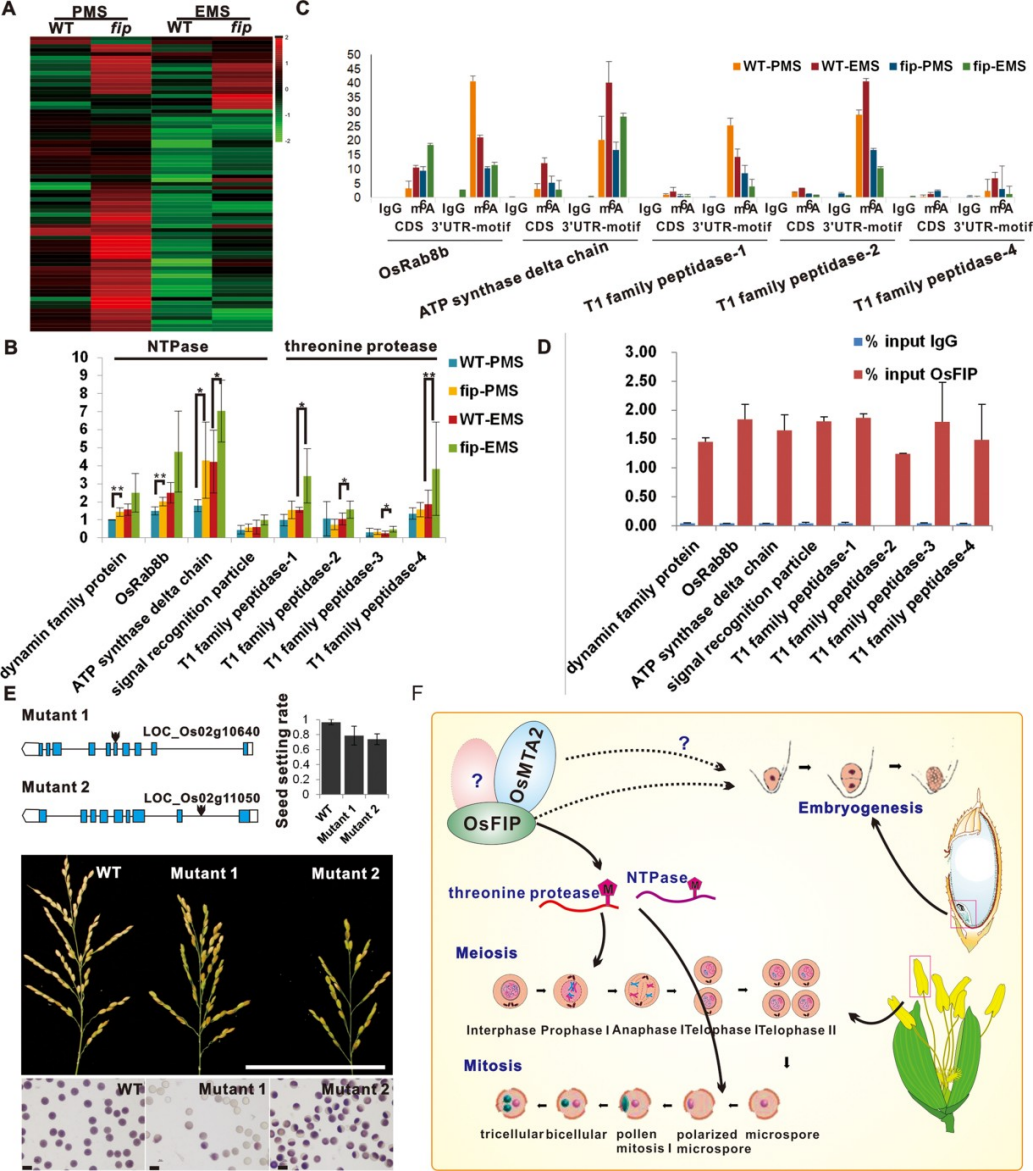
OsFIP regulates the m6A modification and expression by binding to the rice panicle motif. (**A**) Expression pattern of NTPase or threonine protease genes in WT and *fip* panicles during PMS and EMS. (**B**) qRT-PCR validation of eight differentially m^6^A modified NTPase or threonine protease genes in WT and *fip* panicles during PMS and EMS. Values shown are the means ± s.d. (n = 3 biological replications). Significant differences were identified using Student’s *t*-test. (**C**) m^6^A-IP-qPCR of five NTPase or threonine protease genes at the CDS regions without a motif and at the 3’ UTR regions with a motif in WT and *fip* panicles during PMS and EMS. (**D**) OsFIP-RIP-qPCR of eight NTPase or threonine protease genes using Flag antibody in the OXFIP-Flag plants. (**E**) The panicle and pollen grain phenotypes and seed setting rates of two T-DNA insertion mutants of two threonine protease genes. The red arrows indicate the T-DNA insertion site. Scale bars for panicles, 10cm; Scale bars for pollen grains, 100μm. (**F**) Proposed model describing the roles of OsFIP and OsMTA2 in regulating sporogenesis and embryo development.

To analyze how OsFIP affects sporogenesis through m^6^A modification, we then performed Gene Ontology (GO) analysis of the genes that were differentially modified between *fip* and WT panicles during sporogenesis. Consistent with the phenotypes during meiosis, almost no GO terms related to meiosis could be enriched, indicating that OsFIP is not essential for meiosis (**Fig 4G** and **S5 Fig)**. Interestingly, these genes were specifically enriched in threonine protease and NTPase GO terms (**Fig 4G** and **S5 Fig)**. Threonine protease is one of the seven types of proteolytic enzymes. We then analyzed the gene expression patterns and splicing patterns of the differentially modified threonine protease and NTPase genes, and most of them are upregulated in the *fip* samples (**Fig 5A**), but their splicing patterns are only slightly affected (4%), indicating that m^6^A modifications mediated by OsFIP negatively regulate threonine protease and NTPase gene expression. qRT-PCR further confirmed these expression patterns (**Fig 5B)**. However, the gene expression fold change was higher in the *fip*-EMS sample than in the *fip*-PMS sample. We speculated that the higher fold change in the EMS stage might be caused by a greater m^6^A modification change between WT and *fip* RNAs at the EMS stage. Thus, we performed m^6^A dot blot in WT and *fip* panicles in these two stages. The results showed that compared with the m^6^A modification level in WT, the modification level was decreased more significantly in *fip* at the EMS stage than that at the PMS stage (**S3B Fig**), which might be the reason why the fold change of gene expression was higher in the *fip*-EMS sample.

To confirm the m^6^A peak position in these threonine protease and NTPase genes as OsFIP targets at PMS and EMS, we further performed m^6^A-RIP-qPCR in WT and *fip* plants (**Fig 5C)**. Except for two of them that were undetected, five of the other six genes were shown to have m^6^A modifications at the predicted 3’UTR rice panicle specific motif, which were diminished in *fip* plants but not in the gene body region (**Fig 5C)**. We also identified the direct binding of OsFIP to these genes by performing OsFIP-RIP assays in the OXFIP plants using anti-FLAG antibody (**Fig 5D)**. The results showed that all of these genes were bound by OsFIP (**Fig 5D)**. These results indicate that OsFIP directly binds to threonine protease and NTPase genes and mediates their m6A modification at the rice panicle specific motif.

Proteases have been reported to be important for sporogenesis through inducing the apoptosis-like programmed cell death (PCD) of microspores in plants, although the role of threonine protease has not been reported in plants. NTPase has also been reported to affect cytoplasmic male sterility in rice. To verify the roles of these differentially m^6^A modified and expressed NTPases and threonine proteases on sporogenesis, we chose five NTPase genes (LOC_Os02g11050, LOC_Os02g10640, LOC_Os09g32800, LOC_Os03g50520 and LOC_Os12g44150) that are m^6^A modified in an OsFIP dependent way. We obtained their mutant plants from the RMD rice mutant database. Insertion mutant of the two genes LOC_Os02g11050 and LOC_Os02g10640 have abnormal pollen grains, showing the probable role of NTPase genes on sporogenesis (**S4U and S4V Fig)**. Thus, we concluded that OsFIP is essential for threonine protease and NTPase gene expression and/or splicing, which then prevents microspores from early PCD at the early microspores stage.

In summary, *OsFIP* and *OsMTA2* were revealed as the major components of the m^6^A methyltransferase complex in rice, and both proteins interact with each other and they are required for rice reproductive development. *OsFIP* is essential for early sporogenesis by mediating m^6^A modification of a set of threonine protease and NTPase genes. We also reported the characteristics of rice m^6^A modification during sporogenesis in both WT and *fip* plants, and identified a rice sporogenesis stage specific m^6^A modification motif “UGWAMH”. The proposed functions of rice *OsFIP* are shown in **Fig 5E**. This is the first study to report that the m^6^A RNA methyltransferase complex plays an essential role in plant sporogenesis.

## Discussion

Identification of the methyltransferase complex for catalyzing m^6^A formation in RNA is essential for understanding the functions of m6A modification. In mammals, the core components of the complex are METTL3, METTL14 and WTAP. In Arabidopsis, the orthologs of METTL3, METTL14 and WTAP have been identified as AtMTA, AtMTB and AtFIP37. In this study, we characterized the core components of the rice m^6^A methyltransferase complex and demonstrated that OsFIP and OsMTA2 are orthologs of METTL3 and WTAP. Knocking out of OsFIP or OsMTA2 decreased m^6^A mRNA methylation and resulted in sterile phenotypes. Moreover, OsFIP and OsMTA2 interact with each other in the nucleus. We predicted the homologous genes of Arabidopsis AtMTB and mammalian METTL14 and found that OsMTA1, OsMTA3 and OsMTA4 are highly homologous with AtMTB and METTL14. However, OsMTA1, 3 and 4 do not affect m^6^A methylation levels; moreover, they do not interact with OsMTA2 or OsFIP. These results suggested that these proteins might not be the subunits of the rice m^6^A methyltransferase complex. Additional studies are needed to demonstrate whether plant m^6^A methyltransferases have other components.

Most of the molecular functional studies of the plant m^6^A methyltransferase complex have been performed in Arabidopsis. AtMTA and AtFIP37 regulate embryo development, and AtFIP37 determines shoot stem cell fate in Arabidopsis. The ortholog of METTL14, MTB, which is involved in root development, was identified as a component required for m^6^A in Arabidopsis [9]. In this study, we uncovered novel functions of OsFIP in rice. OsFIP regulates pollen development by affecting the m^6^A modification of threonine protease and NTPase genes, which has not been reported for WTAP in mammals or AtFIP37 in Arabidopsis. OsFIP is important for normal sporogenesis progress in rice, and the complete deletion of OsFIP causes early degeneration of pollen grains during the vacuolated pollen stage. A small portion of the *fip* MMCs also has abnormal chromosome distribution and the vacuolated cytoplasm at meiosis prophase I.

The m^6^A-Seq of WT and *fip* panicles showed that OsFIP is indispensable for m^6^A modification of threonine protease and NTPase genes during early sporogenesis. Threonine protease was first described in 1995 in animals, but no study has been performed on plant threonine protease. Interestingly, other kinds of proteases are closely related to sporogenesis. For example, OsCP1 is a rice cysteine protease that is essential for early microspores development [20]. A36 and A39 are two aspartic proteases in Arabidopsis that affect pollen apoptosis-like PCD [21]. It is intriguing to speculate about the specific roles of threonine protease during early sporogenesis under the regulation of m^6^A modification. Many NTPases, especially ATPases, were also regulated by m^6^A modification mediated by OsFIP during sporogenesis. ATPases are well known to be involved in energy metabolism and cytoplasmic male sterility [22–25]. It is possible that m^6^A modification is also important for cytoplasmic male sterility by affecting ATPases during sporogenesis. We also showed that two NTPase genes might function during sporogenesis. This report is the first to describe that the homolog of WTAP regulates the early sporogenesis process.

It is worth mentioning that the roles of OsMTA2 in rice reproductive development were different from those of OsFIP. We observed that the mutation or overexpression of OsMTA2 leads mainly to aborted seeds, which is conserved in Arabidopsis and that the loss of function of AtMTA leads to embryo-lethal phenotypes. These differences between the functions of OsFIP and of OsMTA2 imply that there might be other methyltransferases that interact with OsFIP to modify the mRNAs of gametogenesis-related genes or imply that OsFIP might play an independent role in gametogenesis. Whether WTAP or AtFIP37 have similar roles needs to be further studied.

## Materials and methods

### Plant growth conditions and generation of transgenic rice plants

The growth conditions and generation of transgenic plants were conducted according to Zhang et al. [26]. Briefly, the Zhonghua 11 (*Oryza sativa japonica*) rice cultivar was used in these experiments. Rice plants were grown in the field in Guangzhou, China (23°08′ N, 113°18′ E), where the growing season extends from late April to late September. The average low temperature range is 22.9–25.5°C, and the average high temperature range is 29.7–32.9°C. The day length ranged from 12 to 13.5 h. The plants were maintained with routine management practices. As ZhongHua 11, a japonica variety usually cultivated in Northern part of China, when grown in Gruangdong Province, it flowers earlier and has less tiller numbers and grain numbers. *OsFIP* and *OsMTA1/2/3*,*4* were overexpressed under the control of the CaMV35S promoter. Three overexpression lines with higher expression level than WT plants of these genes respectively were used for the next phenotype analysis. The *OsFIP* and *OsMTA2* knockout mutants were generated using CRISPR-Cas9-based genome editing technology as previously described [27]. T1, T2 and T3 generations of *fip* plants and T3 generation of *mta2* plants were used to analyze the phenotypes. The heterozygous *fip* plants were harvested and used to screen homozygous *fip* plants in the next generation, as homozygous *fip* plants are almost completely sterility. The phenotypes of T1, T2 and T3 generations of *fip* and *mta2* plants are stable. See Supplementary Methods for details.The following primers were used: *OsFIP* Target site: 5’-GTTGGACGTTTTCGCTTCCAAGA-3 and 5’- AAACTCTTGGAAGCGAAAACGTC -3’; *OsMTA2* Target site 1: 5’- GCCGCGGATTCTGGCAGCTCCTTG -3’ and 5’- AAACCAAGGAGCTGCCAGAATCCG -3’; *OsMTA2* Target site 2: 5’- GTTGCCCCCCTCTGAGACCGATGC -3’ and 5’- AAACGCATCGGTCTCAGAGGGGGG -3’; *OsMTA1* Target site: 5’ GCCGTACGGGAGATAACTCAAGGG-3’ and 5’-AAACCCCTTGAGTTATCTCCCGTA-3’; *OsMTA3* Target site:5’-GCCGAAAGGTGATAGACCCTCCAG-3’ and 5’AAACCTGGAGGGTCTATCACCTTT-3’; *OsMTA4* Target site:5’-GCCGAGATTGTCCGACGGGTACA-3’ and 5’-AAACTGTACCCGTCGGACAATCT-3’.

### BiFC and yeast two-hybrid assays

Two-week-old rice shoots were used to isolate protoplasts. A bundle of rice plants (approximately 30 seedlings) were cut together into approximately 0.5-mm strips with propulsive force using sharp razors. The strips were incubated in an enzyme solution (1.5% cellulose RS, 0.75% macerozyme R-10, 0.6 M mannitol, 10 mM MES, pH 5.7, 10 mM CaCl_2_ and 0.1% BSA) for 4–5 h in the dark with gentle shaking (40–50 rpm). After the enzymatic digestion, an equal volume of W5 solution (154 mM NaCl, 125 mM CaCl_2_, 5 mM KCl and 2 mM MES, pH 5.7) was added, followed by shaking (60–80 rpm) for 30 min. Protoplasts were released by filtering through 40-μm nylon mesh into round bottom tubes, followed by washing 3–5 times with W5 solution. The pellets were collected by centrifugation at 800 rpm for 3 min in a swinging bucket. After washing once with W5 solution, the pellets were then resuspended in MMG solution (0.4 M mannitol, 15 mM MgCl_2_ and 4 mM MES, pH 5.7) at a concentration of 2×10^6^ cells mL^-1^. BIFC PEG-mediated transfections were performed as previously described [28]. Protoplasts were observed using a confocal laser-scanning microscope (Zeiss 7 DUO NLO) at 488 and 561 nm excitation. All manipulations described above were performed at room temperature.

### m^6^A dot blot assay

m^6^A dot blot assay was performed as previously described[29] with some modifications. Briefly, total RNA was isolated from panicles of different transgenic lines with RNAiso plus (TAKARA) according to the manufacturer's instructions. The RNA samples were loaded to the nylon membrane and UV crosslinked to the membrane. Then the membrane was stained with 0.02% methylene blue (sigma) (in 0.3M NaAc, PH 5.5). After the staining, the membrane was washed by 0.5% SDS and TBST, and then blocked with 5% nonfat dry milk (in 1X TBST) for 1 hours and incubated with a specific anti-m6A antibody (1:5000 dilution, Abcam) overnight at 4°C. Then the HRP-conjugated goat anti-rabbit IgG (Santa Cruz Biotechnology) was added to the blots for 1 hour at room temperature and the membrane was developed with Amersham ECL Prime Western Blotting Detection Reagent (Milipore).

### DAPI staining

The 4',6-diamidino-2-phenylindole (DAPI) staining was performed as previously described, with minor modifications [30]. The fixed tissue was washed twice with water and twice with 10 mM citrate buffer, pH 4.5. Four to six anthers were placed in a small drop of 60% acetic acid on a slide and pressed with another slide to release microspore mother cells. The slides were then separated, and the samples were dried at room temperature for 5 min. A total of 5 μL DAPI solution (1 μg/mL DAPI in a buffer with 50% glycerol and 10 mM citrate, pH 4.5) was placed onto the slide, covered with a cover glass and sealed with clear nail polish. The slides were examined under a fluorescence microscope (Leica DM5000B).

### Eosin B staining

Eosin B staining was performed previously described [31]. The ovaries were dissected in 70% ethanol under a binocular dissecting microscope, and sequentially hydrated in 50% ethanol, 30% ethanol and distilled water. After that, the ovaries were pretreated in 2% aluminium potassium sulphate for 20 min. The ovaries were then stained with 10 mg/l of eosin B solution for 10–12 h at room temperature. The samples were post-treated in 2% aluminium potassium sulphate for 20 min and rinsed three times with distilled water, followed by dehydration with a series of ethanol solutions (30%, 50%, 70%, 90% and 100%). Subsequently, the dehydrated samples were transferred to a mixture of absolute ethanol and methyl salicylate (1:1) for 1 h and then cleared in pure methyl salicylate solution for at least 1 h. The slides were examined under a confocal laser scanning microscope (Zeiss 7 DUO NLO).

### Semi-thin sections for light microscopy of anthers

The samples were fixed in 2.5% paraformaldehyde—3.0% glutaraldehyde in 0.1 mol/L PBS (pH 7.2) for 4 h at 4°C and then washed 3 times in the same buffer, which was followed by post-fixation in 1% osmium tetroxide for 2 h at room temperature and 3 rinses using the same buffer. Specimens were dehydrated in a graded ethanol series and embedded in Epon812 (SPI Supplies Division of Structure Probe Inc., West Chester, PA, USA). Polymerization took place for 24 h at 40°C, which was followed by 24 h at 60°C. Specimens were cut to a thickness of 1 μm on a Leica RM2155 and were stained with 0.5% toluidine blue. Sections were observed and photographed with a Leica DMLB microscope.

### Examination of gene expression by qRT-PCR analysis

Total RNAs from rice seedlings at 14 d after germination or panicles before heading were reverse transcribed using the PrimeScript RT reagent kit (Takara, Japan). Real-time PCR was performed using SYBR Premix Ex Taq (Takara, Japan) to detect the PCR products. *Actin2* was used as the reference gene. Real-time PCR was performed according to the manufacturer’s instructions (Takara, Japan), and the resulting melting curves were visually inspected to ensure the specificity of the product detection. Gene expression was quantified using the comparative Ct method. The experiments were performed in triplicate, and the results are represented as the mean ± s.d. For *Actin2*, the primers were Actin2-F (5’-GTGCTTTCCCTCTATGCT-3’) and Actin2-R (5’-CTCGGCAGAGGT GGTGAA-3’); and for *OsFIP*, the primers were OsFIP-F (5-GGAAGAAAGTGCGCCAGGTG-3) and OsFIP-R (5- GATTTGGCAGCCTCCCGTTC -3); for *OsMTA2*, the primers were OsMTA2-F (5’-AGGTGGTTCCCAGCTGAAGG-3’) and OsMTA2-R (5’-GCAGGTCTTTGTGTGACGGC-3’); for *MEL1*, the primers were MEL1-F (5’-GCTATACCTATGCGCGATG-3’) and MEL1-R (5’-ATCCGAACTCTCTCCTTCCA-3’); for *MEL2*, the primers were MEL2-F (5’-TGTGATGCAGCTTGTCCCAT-3’) and MEL2-R (5’-CGCTCCATGACTCCCACATA-3’); for *SPO11-4*, the primers were SPO11-4-F (5’-CAATGCGAATCAGCGGGAAG-3’) and SPO11-4-R (5’-TCAATCCAGCCCCAAGTGTC-3’); for *PAIR1*, the primers were PAIR1-F (5’-AAAGGTGGAGCAGGGAAAGG-3’) and PAIR1-R (5’-TGCTGACTGGTGCCTTCTTT-3’); for *RPA2C*, the primers were RPA2C-F (5’-CAGCACCGGGAAGATCCCAC-3’) and RPA2C-R (5’-TGCAGGGGGTAGTCCTTGGT-3’); for *CRC1*, the primers were CRC1-F (5’-AGGGTGGCAATTCTCTCTGG-3’) and CRC1-R (5’-ATCAAAGCGTGCAGGAAAGC-3’); for *ZIP4*, the primers were ZIP4-F (5’-ACTCTCTTCACCGAAGCACT-3’) and ZIP4-R (5’-CTTGAGCCCCTCTAGATTTG-3’); for *Rec8*, the primers were Rec8-F (5’-TCCGGAAGGTCCAAGAGGCA -3’) and Rec8-R (5’-TGAGTTGCTAAAACGCATGCTTGA-3’).

### In situ hybridization

RNA in situ hybridization was performed as previously described, with minor modifications [32]. Briefly, the plant materials were fixed in FAA fixative for 8 h at 4°C after vacuum infiltration and dehydrated using a graded ethanol series, followed by a xylene series, and embedded in Paraplast Plus (Sigma-Aldrich). Microtome sections (9 μm) were mounted on Probe-On Plus microscope slides (Fisher). The 141-bp regions of *OsFIP* was amplified using the primers 5’- GGAAGAAAGTGCGCCAGGTG -3’ and 5’- GATTTGGCAGCCTCCCGTTC -3’ and then subcloned into the pEASY-T3 (TransGen Biotech) vector and used as the template to generate sense and antisense RNA probes. The *OsMTA2* probe was amplified using the primers 5’- AGGTGGTTCCCAGCTGAAGG -3’ and 5’- GCAGGTCTTTGTGTGACGGC -3’. The antisense probe was transcribed using T7 RNA polymerase, and the sense probe was synthesized using SP6 RNA polymerase. Digoxigenin-labeled RNA probes were prepared using a DIG RNA Labeling Kit (SP6/T7) (Roche) according to the manufacturer's instructions. Photomicrographs were obtained using a bright-field microscope (Leica DM5000B).

### m^6^A - Sequencing

The panicles at the pollen mother cell meiosis stage (2–5 mm spikelet) and the early microspore stage (7–8 mm spikelet) from the wild-type and *fip* plants (n > 20 plants for each sample) were collected to extract the total RNA. Two biological replicates of m6A RIP sequencing were performed for the two WT samples, but only one replicate were performed for fip samples because of the insufficient samples of fip plants. m^6^A sequencing was performed as previously described with modifications using ant- m^6^A antibody (Synaptic Systems, cat. No. 202003) [33]. The RNA-seq was performed on the Illumina Hiseq 2500 platform.

The m^6^A modification peaks were called with the exomePeak program with strict criteria (false discovery rate (FDR) <0.05, *P*-value <0.01and fold change (FC)>2). The *de nov*o motif identification of the m6A peak data was performed by susing the HOMER software to obtain their position weight matrices and accurate motif regions. We assigned all modification sites to gene regions covering CDS, 3’UTR, 5’ UTR, intron and exon region. The gene expression level was calculated using RPKM method (Read Per kb per Million reads). The differentially expressed genes were then screened. Gene Ontology (GO) enrichment analysis was performed to decipher the biological processes involving the differentially modified genes.

### m^6^A -IP-qPCR

m^6^A-IP-qPCR was performed using the magna RIP kit (Millipore, 2982054). 50μg total RNA was used, after treated with DNaseⅠNthermo, EN0525), the RNA was fragmented by 0.1 M ZnCl2 at 94℃ for 100 s then immunoprecipitated with 3 μg anti-m^6^A antibody (Synaptic Systems, 202003). RNAs isolated were analyzed by RT-PCR.

### Biochemistry assay for m^6^A methyltransferase activity *in vitro*

The *in vitro* methyltransferase activity assay was performed in a standard 50 μL of reaction mixture containing the following components: 0.15 nmol RNA probe, fresh purified FIP or MTA proteins, 0.8 mM *d*_*3*_-SAM, 80 mM KCl, 1.5 mM MgCl_2_, 0.2 U μL^−1^ RNasin, 10 mM DTT, 4% glycerol and 15 mM HEPES (pH 7.9). The reaction was incubated at 16°C for 12 h. The methylation of RNA-probe was measured by immunoblotting with the m6A antibody.

### Biotinylated RNA probes pulldown assay

The pulldown assay was performed using the Pierce Magnetic RNA-Protein Pull-Down Kit (thermo scientific, 20164) according to its instruction. Cells were lysed in 200 μl of lysis buffer (150 mM KCl, 25 mM Tris pH 7.4, 0.5 mM DTT, 0.5% NP40, with 1 nM PMSF) in 4°C for 1h. Add 50 pmol of RNA probe to 50 μL of streptavidin magnetic beads, then incubated the tube for 30 minutes at room temperature with rotation. After washed with 50μL 20mM Tris (pH 7.5) twice, the RNA-bound beads were incubated with the lysate for 1 h at 4°C with rotation. Beads were washed three times with 500 μl wash buffer (20mM Tris (pH 7.5), 10mM NaCl, 0.1% Tween-20 Detergent). Finally, beads were boiled for 10 min in SDS sample buffer, and followed by Western blotting analysis.

## Supporting information

S1 Fig Conservation analysis of *OsFIP* and *OsMTA1/2/3/4*. The functional regions are indicated by black boxes. (**A**) Conservation analysis of *OsMTA2*. (**B**) Conservation analysis of *OsFIP*. (**C**) Conservation analysis of *OsMTA1/3/4*.
(JPG)

S2 Fig Genome editing types of the transgenic plants. (**A**) Editing types of the heterozygous *OsMTA2* knockout plants. (**B**) Editing types of the homozygous and heterozygous *OsFIP* knockout plants. (**C**) Editing types of the heterozygous *OsMTA1*, *3*, and *4* knockout plants.
(JPG)

S3 Fig Dot blot analysis of RNA m6A levels, the interactions between OsFIP and OsMTAs and phenotype analysis of transgenic plants. (**A**) Dot blot analysis of RNA m^6^A levels in wild-type, *fip*, *mta2*, OXFIP and OXMTA2 panicles. MB, methylene blue staining (as loading control). (**B**) Dot blot analysis of RNA m^6^A levels in wild-type and *fip* panicles at PMS and EMS stage. MB, methylene blue staining (as loading control). (**C**) Dot blot analysis of RNA m^6^A levels in wild-type, *mta1*, *mta3* and *mta4* seedlings. (**D-E**) OsMTA2 interacts with OsFIP in both rice nuclei (**D**) and yeast (**E**), Scal bar, 2μm. (**E**) Yeast two-hybrid between OsMTA1, 3, 4 and OsFIP and OsMTA2. (**F**) The morphology of WT and *fip* plants during vegetable stage. (**G**) Tiller number per plants of WT and the transgenic plants. Values shown are the means ± s.d. (n > 20 plants). Significant differences were identified using Student’s *t*-test. (**H**) embry sacs of WT, *fip* and *mta2* plants before flowering or 21 Days after flowering. (**I**) Transverse semithin sections of homozygous *fip* anthers at stages 5, 6 7 and 8 from left to right panels. (**J**) Transverse semithin sections of heterozygous *fip* anthers at stages 12. SDS, sporogenous cells differentiation stage; PMC, pollen mother cell; MSI, meiosis I; MSII, meiosis II; MPS, mature pollen stage.
(JPG)

S4 Fig Histological analysis of WT and *fip* meiosis and sporogenesis in anthers, and the effect of OsFIP on gene expression and splicing, as while as the roles of threonine protease in sporogenesis. (**A**) Frequency of MMCs at various meiotic stages in anthers ranging from 0.3–0.8 mm in length. Gray and red bars indicate the frequency of WT and heterozygous *fip* MMCs at various stages. (**B-P**) The meiosis processes of WT (**B-H**), heterozygous *fip* (**I-K**) and homozygous *fip* (**L-P**) MMCs. The arrows indicate the chromosome bridge and chromosome fragments. Scale bars, 4 μm. (**R**) The number of nucleus of WT and *fip* microspores during late micropore stage (LMS), late binucleate pollen stage (LBPS) and mature pollen stage (MPS). mmc, microspore mother cells; nu, nucleus; no, nucleolus; cc, condensed chromosome; m, mitochondria; v, vacuoles. (**Q**) The microspores of WT and *fip* plants during late microspore stage (LMS), late binucleate pollen stage (LBPS) and mature pollen stage (MPS). Red scircles indicate the nucleus. Scale bars, 20 μm. (**S**) Expression pattens of genes which are m^6^A modified in a OsFIP dependent way. (**T**) Splicing patterns of genes which are m^6^A modified in a OsFIP dependent way.
(JPG)

S5 Fig GO analysis of the differentially modified genes in *fip* panicles. (PNG)
