# Supplementary figures and images for: The subunit of RNA N6-methyladenosine methyltransferase OsFIP regulates early degeneration of microspores in rice
Source: PLoS Genet. 2019 May 22;15(5):e1008120. doi: 10.1371/journal.pgen.1008120 (PMC6548400; doi:10.1371/journal.pgen.1008120)

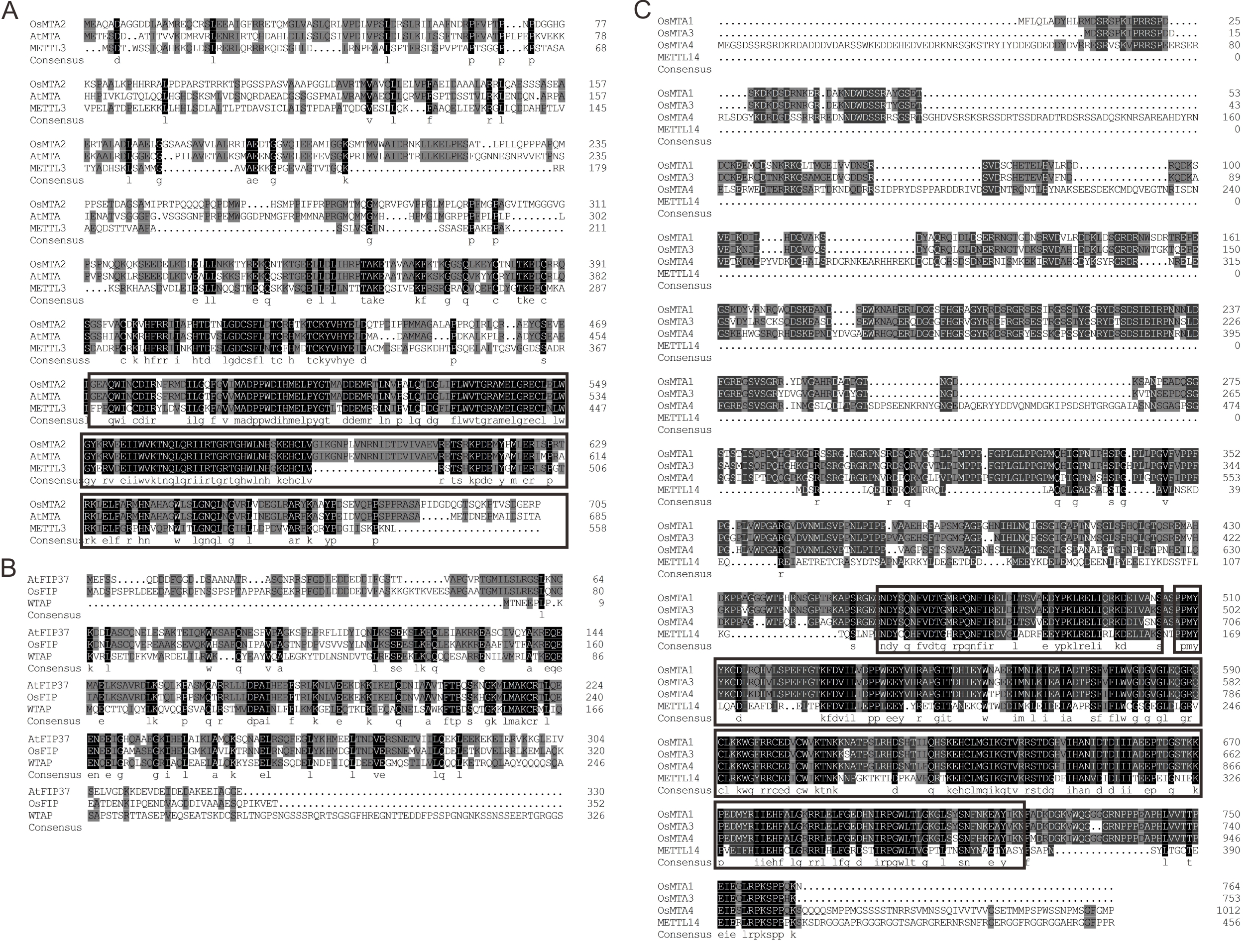

Supplement: S1 Fig — The functional regions are indicated by black boxes. (A) Conservation analysis of OsMTA2. (B) Conservation analysis of OsFIP. (C) Conservation analysis of OsMTA1/3/4. (JPG) [file pgen.1008120.s001.jpg]

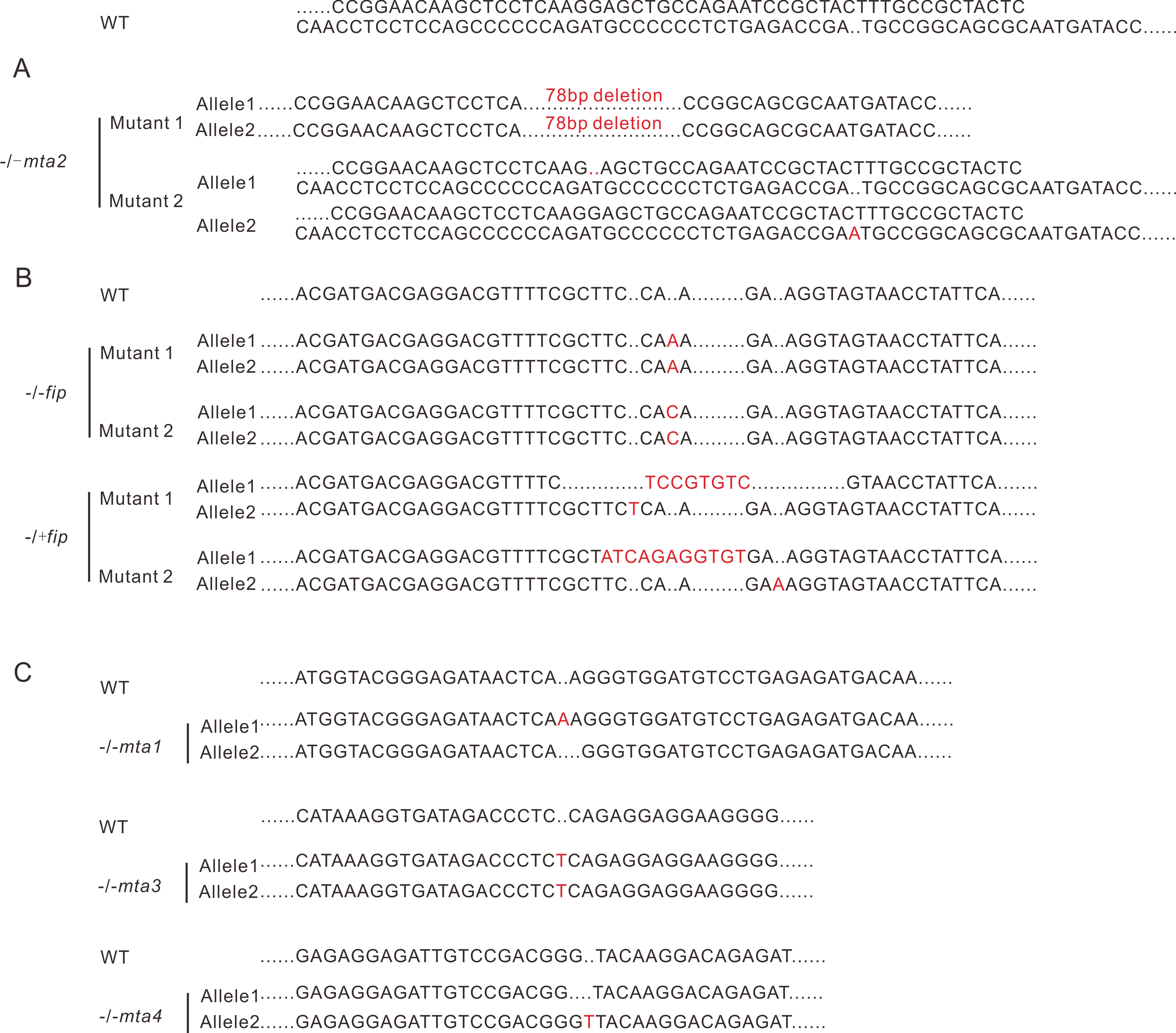

Supplement: S2 Fig — (A) Editing types of the heterozygous OsMTA2 knockout plants. (B) Editing types of the homozygous and heterozygous OsFIP knockout plants. (C) Editing types of the heterozygous OsMTA1, 3, and 4 knockout plants. (JPG) [file pgen.1008120.s002.jpg]

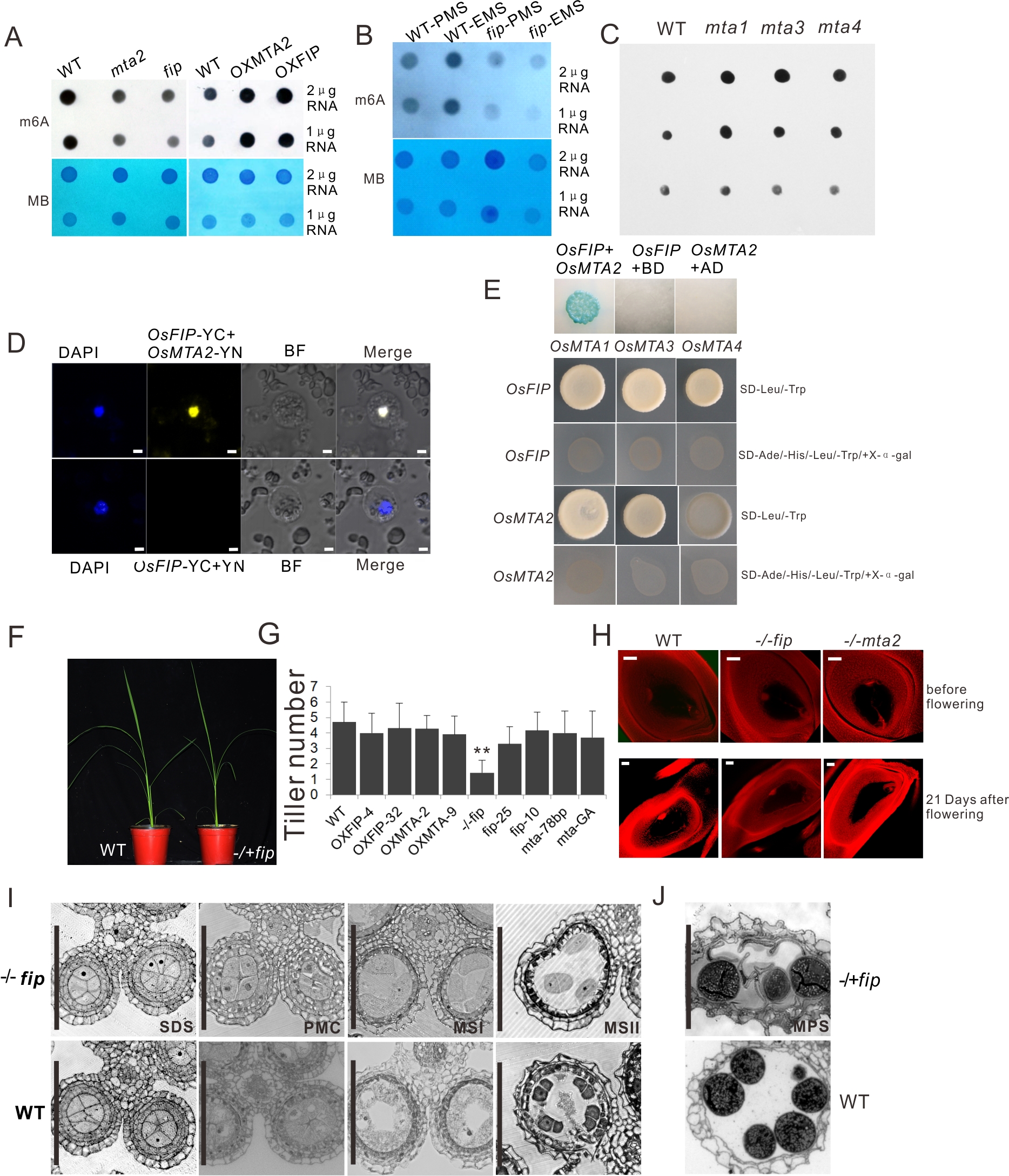

Supplement: S3 Fig — (A) Dot blot analysis of RNA m6A levels in wild-type, fip, mta2, OXFIP and OXMTA2 panicles. MB, methylene blue staining (as loading control). (B) Dot blot analysis of RNA m6A levels in wild-type and fip panicles at PMS and EMS stage. MB, methylene blue staining (as loading control). (C) Dot blot analysis of RNA m6A levels in wild-type, mta1, mta3 and mta4 seedlings. (D-E) OsMTA2 interacts with OsFIP in both rice nuclei (D) and yeast (E), Scal bar, 2μm. (E) Yeast two-hybrid between OsMTA1, 3, 4 and OsFIP and OsMTA2. (F) The morphology of WT and fip plants during vegetable stage. (G) Tiller number per plants of WT and the transgenic plants. Values shown are the means ± s.d. (n > 20 plants). Significant differences were identified using Student’s t-test. (H) embry sacs of WT, fip and mta2 plants before flowering or 21 Days after flowering. (I) Transverse semithin sections of homozygous fip anthers at stages 5, 6 7 and 8 from left to right panels. (J) Transverse semithin sections of heterozygous fip anthers at stages 12. SDS, sporogenous cells differentiation stage; PMC, pollen mother cell; MSI, meiosis I; MSII, meiosis II; MPS, mature pollen stage. (JPG) [file pgen.1008120.s003.jpg]

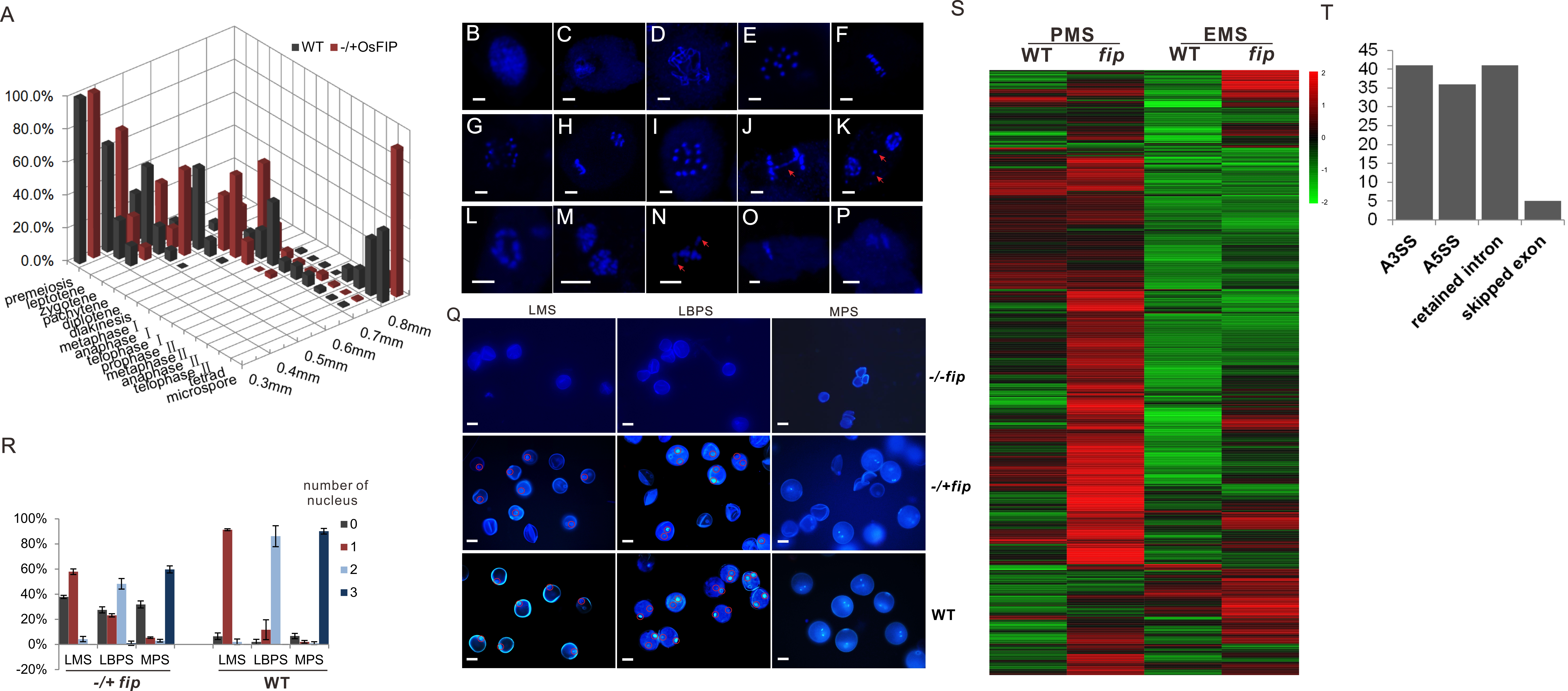

Supplement: S4 Fig — (A) Frequency of MMCs at various meiotic stages in anthers ranging from 0.3–0.8 mm in length. Gray and red bars indicate the frequency of WT and heterozygous fip MMCs at various stages. (B-P) The meiosis processes of WT (B-H), heterozygous fip (I-K) and homozygous fip (L-P) MMCs. The arrows indicate the chromosome bridge and chromosome fragments. Scale bars, 4 μm. (R) The number of nucleus of WT and fip microspores during late micropore stage (LMS), late binucleate pollen stage (LBPS) and mature pollen stage (MPS). mmc, microspore mother cells; nu, nucleus; no, nucleolus; cc, condensed chromosome; m, mitochondria; v, vacuoles. (Q) The microspores of WT and fip plants during late microspore stage (LMS), late binucleate pollen stage (LBPS) and mature pollen stage (MPS). Red scircles indicate the nucleus. Scale bars, 20 μm. (S) Expression pattens of genes which are m6A modified in a OsFIP dependent way. (T) Splicing patterns of genes which are m6A modified in a OsFIP dependent way. (JPG) [file pgen.1008120.s004.jpg]

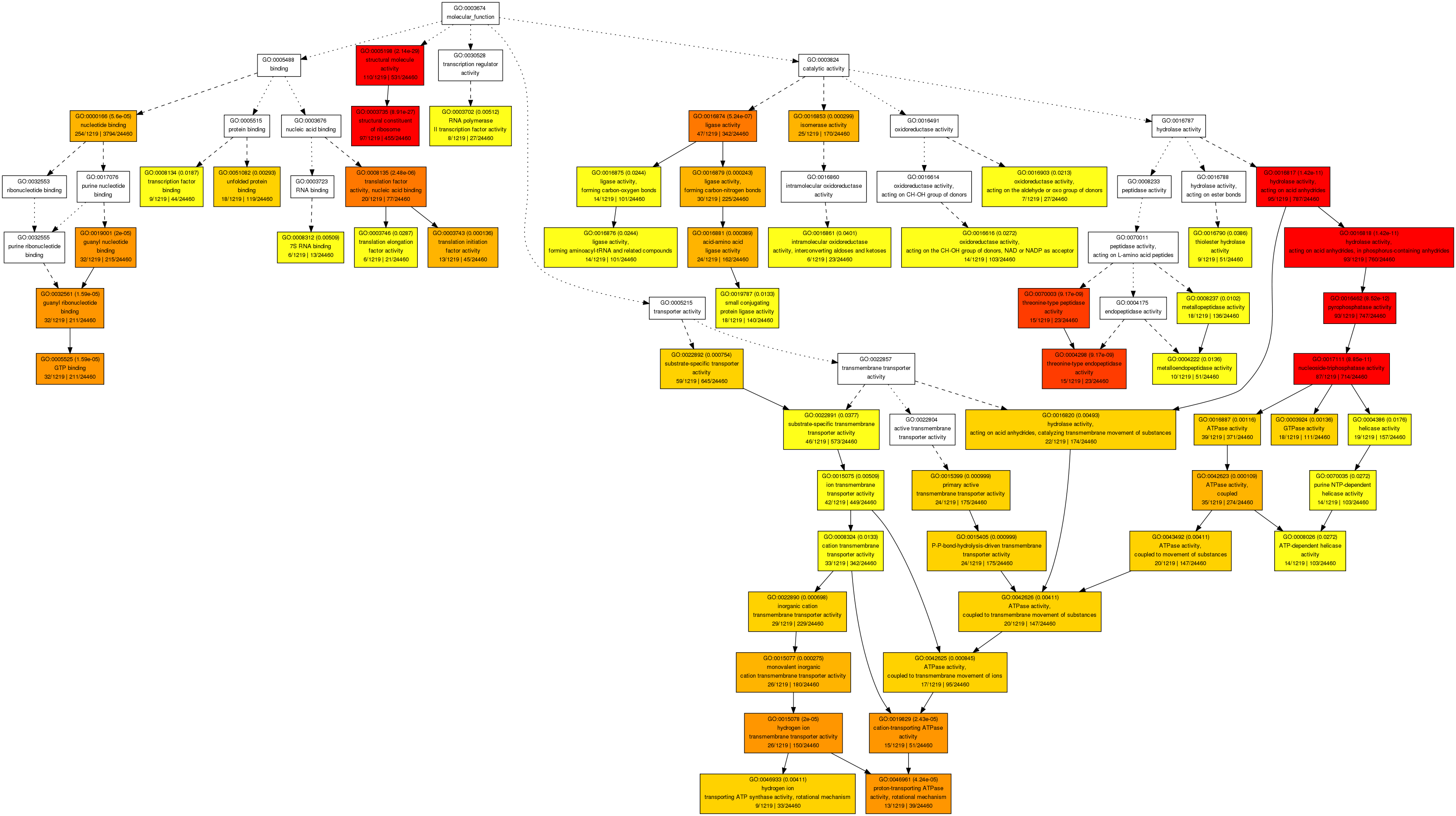

Supplement: S5 Fig — (PNG) [file pgen.1008120.s005.png]
